# The interplay between the gut-brain axis and the microbiome: A perspective on psychiatric and neurodegenerative disorders

**DOI:** 10.3389/fnins.2022.1030694

**Published:** 2022-10-28

**Authors:** Yasir Bashir, Asad U. Khan

**Affiliations:** Medical Microbiology and Molecular Biology Lab, Interdisciplinary Biotechnology Unit, Aligarh Muslim University, Aligarh, Uttar Pradesh, India

**Keywords:** gut microbiota, microbiota-gut-brain axis, psychiatric diseases, neurodegenerative disorders, therapeutics, microbiome

## Abstract

What is the effect of our gut microbial flora on brain? Does the gut microbiome have any role in the causation of psychiatric and neurodegenerative diseases? Does the effect of gut microbiota traverse the gut-brain axis? Questions like these have captured the interest and imagination of the scientific community for quite some time now. Research in the quest for answers to these questions, to unravel the potential role of the microbiota inhabiting the gut in controlling brain functions, has progressed manifold over the last two decades. Although the possibility of microbiome as a key susceptibility factor for neurological disorders viz. Parkinson’s disease, Alzheimer’s disease, multiple sclerosis, and autism spectrum disorder has bolstered by an increase in the clinical and preclinical evidence, the field is still in its infancy. Given the fact that the diversity of the gut microbiota is affected by various factors including the diet and exercise, the interpretation of such data becomes all the more difficult. Also, such studies have been mostly conducted on animal models, so there is a need for randomized controlled trials in human subjects, corroborated by longitudinal studies, to establish if modulating the gut microbiota can unravel novel therapeutic interventions. Exploring the genomic, metagenomic and metabolomic data from clinical subjects with psychiatric and neurological diseases can prove to be a helpful guide in individual treatment selection.

## Introduction

The gut microbiota, the collection of all microbes inhabiting the gastrointestinal (GI) tract, is an integral part of human body. Gut microbiome is the total genetic pool of the microbiota inhabiting the human gut. The number of microbes in the gut exceeds 10^14^ and it harbors bacteria, the most abundant, extensively studied and well characterized gut microbes, archaea, yeasts, protozoa, viruses, parasites such as helminths, and single-celled eukaryotes (Cryan et al., 2019). Gut microbiota perform a wide array of physiological functions; they provide protection against pathogens, shape the intestinal epithelium, regulate the host immunity, harvest energy and provide essential nutrients such as vitamins (Thursby and Juge, 2017). The colonization of the gut in the infant commences at birth on exposure to the maternal microbiota during delivery (Perez-Munoz et al., 2017). A number of factors can influence this gut colonization viz. prematurity, mode of delivery, host genetics, the nature of nutrition, environmental stressors, antibiotic exposure, and maternal infection, obesity or stress (Gilbert et al., 2018; Cryan et al., 2019). However, diet is thought to have the greatest influence on the composition of the microbiota inhabiting the gut (Claesson et al., 2012; Sandhu et al., 2017). The gut microbiota, in turn, has been hypothesized to impact the functioning of the host organ system and over the last decade or so, the impact of gut microbiome studies has revolutionized the field of biomedicine with the realization that targeting the gut microbiome can help find novel therapeutic strategies owing to the findings that the gut microbiota and the gut microbiome have a pivotal role in the maintenance of host homoeostasis and in regulating the central nervous system (CNS) as well as major organ systems of the host.

The taxonomic and functional classification of the gut microbiota is essential to understanding the gut microbiome and its possible effects. Of late, the metagenomic shotgun sequencing-based studies have been instrumental in unraveling the composition and dynamics of the gut microbiota. The application of specific bioinformatics tools, in addition to advancements in next-generation sequencing (NGS) technology, has revolutionized the field of gut microbiomics (Wensel et al., 2022). Sequence-based metagenomics has been applied to study diversity of gut microbiota and dysbiosis, as well as its role in disease and health. Function-based metagenomics has identified novel functional genes including antibiotic resistance genes, microbial pathways, functional dysbiosis of the gut microbiota, and unraveled host-microbiota relationship (Wang et al., 2015).

These technologies provide a deeper understanding of the biological properties of gut microbiota and their potential to affect the physiology of the host. Given the fact that most of the microbes in nature are inaccessible as they are unculturable, there is a need to explore culture-independent methods, i.e., metagenomics, metatranscriptomics, microbiomics and metabolomics, to unravel the taxonomy, functional potential, and physiological roles of the unculturable microbes inhabiting the gut.

In this review, we discuss in detail the involvement of gut microbiota in the onset of several neuropsychiatric and neurodegenerative disorders and the scientific progression in this domain.

## Microbiota–Gut–Brain axis

The story about the first documented report of the possible connection between the gut and the brain dates back to the 19th century when the United States Army surgeon William Beaumont was treating a Canadian fur-trader Alexis St. Martin who was accidentally shot at close range. The findings of the study in the course of the treatment opened a window of opportunity, though serendipitous, to take forward the study of physiology of digestion and gut (Beaumont, 1833). The author observed that when his patient became irritable or angry, the rate of digestion got affected greatly, indicating the process of digestion was getting affected by his emotional state, i.e., there is a brain-gut axis (Cryan et al., 2019). Such breakthrough observations were succeeded by other studies that paved the way for the development of animal experimentation models, emphasizing on the critical role of gut-brain axis communication in health and disease homeostatic processes. For example, Ivan Pavlov exteriorized a piece of dog intestine (referred to as the “Pavlov pouch”) to study the physiological process of digestion in dogs making it one of the first documented uses of an animal experimentation model in modern biology (Pavlov, 1897). The advent of brain imaging technologies expedited the accumulation of the scientific evidence in favor of the gut-brain axis and resulted in the emergence of the full appreciation of the bidirectionality (gut-to-brain and brain-to-gut) of this axis, i.e., changes in the gut microbial community affect host behavior and alteration in behavior affects the composition of the microbiota inhabiting the gut (Collins and Bercik, 2009). Over the last 15 years, research in this domain has given rise to a new factor, the gut microbiota, as a key player regulating the gut-brain axis. The two-way functional communication reflects a fundamental role of the gut microbiota and their synergy with the host in accessing signaling pathways pertaining to the gut-brain axis to regulate behavior of the host and functioning of the nervous system. Research on animal models has explored this bidirectionality and current evidence shows that changes in the diversity and functional role of some gut microbiota members is connected with neurophysiological disorders, inferring that the gut microbiota regulates nervous system development emphasizing the idea of a microbiota–gut–brain axis (Mu et al., 2016).

A perturbation in the gut microbiota composition (dysbiosis) leads to the signals being sent to the brain that subsequently manifest as enhanced cellular degeneration, low-grade inflammation, increased oxidative stress, and perturbed energy metabolism (Noble et al., 2017), contributing to the progression of many neuropsychiatric, neurological, and neurodevelopmental conditions, especially neurodegeneration (Friedland, 2015; Mulak and Bonaz, 2015).

**FIGURE 1:**
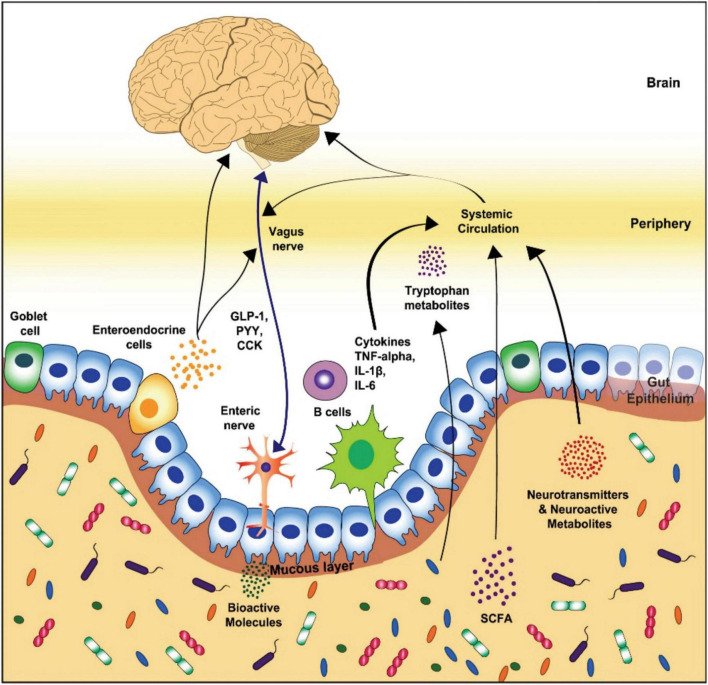
Schematic representation of the key routes of communication in gut-brain axis (Modified from Cryan et al., 2019). GLP-1, glucagon-like peptide-1; PYY, peptide YY; CCK, cholecystokinin; TNF, tumor necrosis factor; IL, interleukin; SCFA, short-chain fatty acid.

The most abundant phyla of the gut microbiota in healthy beings, Bacteroides and Firmicutes (Rinninella et al., 2019), influence the host through the neural-, endocrine, neuroendocrine, and immune pathways (Grenham et al., 2011; Foster and McVey Neufeld, 2013; Naseribafrouei et al., 2014; Dinan and Cryan, 2016; Foster et al., 2017). The neural pathway functions *via* vagus and enteric nerves. On the other hand, the endocrine, neuroimmuno- and neuroendocrine signaling pathways function through systemic circulation. Tryptophan metabolites, vagal nerve, and microbial metabolites such as peptidoglycan or short-chain fatty acids (SCFAs) act as the key routes of communication between the brain and the enteric microbiota (Figure 1; Michel and Prat, 2016; Dinan and Cryan, 2017a; Foster et al., 2017).

The neurotransmitters also play a vital role in this two-way communication. The gut microbiota can affect the brain function by modulating neurotransmitters such as catecholamines (epinephrine, norepinephrine, dopamine), serotonin, glutamine, and γ-Aminobutyric acid (GABA) (Fendt et al., 2008; Winter et al., 2018). The gut microbiota can either produce these neurotransmitters by themselves or influence their synthesis/metabolism. For example, serotonin is produced by *Candida*, *Enterococcus*, *Escherichia*, and *Streptococcus* (Dinan et al., 2015), GABA is generated by *Bifidobacterium* and *Lactobacillus* (Reardon, 2014; Dinan et al., 2015; Dinan and Cryan, 2017b), acetylcholine by *Lactobacillus*, dopamine by *Bacillus* and *Serratia*, while norepinephrine is produced by *Escherichia* and *Saccharomyces* (Lyte, 2011). The blood-brain barrier (BBB) protects the brain from all unwanted pathogens and metabolites, so neurotransmitters produced in the gut have no chance to reach the brain. The only exception being GABA, the major inhibitory neurotransmitter of the host nervous system (Cryan et al., 2019). There are transporters that carry GABA across the BBB while the neurotransmitters produced in the gut act on the ENS and thus have a covert influence on the brain (De Caro et al., 2019; Pascale et al., 2020). In addition, the gut microbiota influence the serotonin amounts in the brain by regulating the amount of tryptophan, a serotonin precursor. This is accomplished by synthesizing enzymes that regulate pathways of tryptophan metabolism, leading to the neuroactive serotonin (Agus et al., 2018). Serotonin has been shown to play a key role in modulating the bacterial motility and inducing the virulence genes expression in bacteria *via* quorum-sensing. More studies are required to unravel the functional dynamics of these neurotransmitters and their importance as mediators of crosstalk between host and gut microbiota.

## The paradigm shift

The monoaminergic systems within the brain have been the most sought-after target for treating the common psychiatric disorders and this has been the most effective pharmacological therapy dating back to the 1950s. Over the past decade or so, the microbiota-gut-brain axis has begun to emerge as a key player in regulating the stress response and maintaining the brain health. So there is a paradigm shift from the traditional modes of pharmacological therapies into the new era of “microbial omics.” The fields of genomics, metagenomics, transcriptomics, microbiomics, metabolomics and proteomics have greatly changed the way we look at things now. The main reason for the gut microbiota to have emerged as such an exciting area in neurological sciences is the advancement in next generation sequencing and metabolomics technologies (Rosario et al., 2020). Reduction in the costs of sequencing has further eased our access to the inner-most details of the microbiome. Owing to such advancements in the technology, gut microbiome has been found to have a key role in neurodevelopment and behavior in preclinical studies (Cryan and Dinan, 2012; Sampson and Mazmanian, 2015), opening up avenues to explore the potential of this microbiota–gut–brain axis to develop novel therapeutic strategies for treating psychiatric conditions such as major depression or anxiety disorders and in the development of novel psychotropics (Dinan et al., 2013).

## Neuropsychiatric diseases and the microbiota-gut-brain axis

### Depression and anxiety

Depression, a stress-related disorder, is one of the most common neuropsychiatric diseases having multifactorial etiology. This worrisome disease has profound influence on the personality of the patient with considerable social consequences. Depression is manifested as the dysregulation of hypothalamic–pituitary–adrenal (HPA) axis which is the most commonly found abnormality in patients with depression and is manifested as elevated levels of corticotropin releasing factor (CRF) and cortisol (Dinan and Cryan, 2016). Additionally, such patients also have an increase in the plasma concentrations of pro-inflammatory cytokines viz. IL-6, TNF, and IL-1β (Farooq et al., 2017).

Gut microbiota exerts a major influence on both the factors involved in depression, i.e., HPA axis as well as the immune system, bolstering the idea that there is a strong connection between the stress response and the gut microbiota (Cryan et al., 2019). In their research on maternally separated mice, De Palma et al. (2015) carried out a comprehensive study about the role of gut microbiota in stress response. They observed that on maternal separation (MSp), an early life stress model, the mice displayed the depression- and anxiety-like behavior in specific pathogen-free (PF; mice that possess normal gut microbiota) but not in germ-free (GF; mice that are entirely devoid of microbiota) conditions. The colonization of the two groups of adult GF mice–MSp and control (which was not MSp) with the PF mice microbiota resulted in differences in the microbiota composition of the two groups. The differences arose despite the fact that both the mice were colonized by the same donor. Also, it was only the MSp mice that displayed the depression- and anxiety-like behavior, emphasizing that stress due to MSp resulted in dysbiosis in the gut, thus making it a critical determinant of abnormal behavior. GF mice also exhibited excessive HPA axis activation in response to stress due to MSp compared to PF mice, compounding the link between stress response and the gut microbiota. The pioneering study of Sudo et al. (2004) was the first to demonstrate that GF mice when stimulated by an acute stressor exhibits an exaggerated HPA axis response.

**FIGURE 2:**
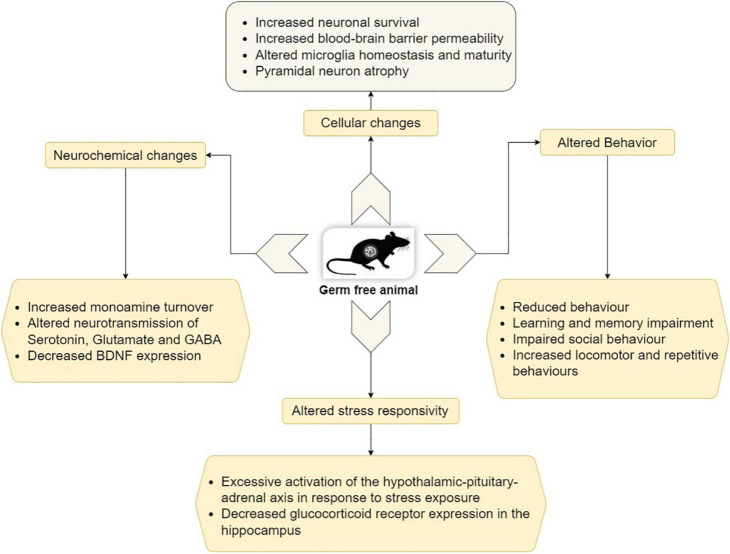
Behavioral, molecular, cellular, and neurochemical changes in GF animals (Modified from Socała et al., 2021).

Germ-free animals differ widely from conventional animals in their behavioral patterns. GF mice display learning and memory impairment, reduced anxiety level, cognitive deficits, as well as impaired social behavior (Diaz Heijtz et al., 2011; Luczynski et al., 2016). Figure 2 depicts the behavioral phenotype and also some molecular, cellular, and neurochemical changes in GF animals.

In order to investigate the potential of using psychobiotics (live bacteria conferring a mental health benefit on the host) as an intervention strategy in depression, the supplementation of gut microbiota viz. *Bifidobacterium longum* and *B. breve* was able to reduce anxiety in an anxious strain of mouse (Savignac et al., 2014). Thus, modulating the gut microbiota for treating the stress-related brain-gut axis disorders may open up new avenues in the field of neurogastroenterology.

Depression and anxiety usually go hand-in-hand, and the studies involving the microbiota-gut-brain axis in depression generally include measures of anxiety and vice versa. The main clinical manifestation in anxiety disorder are persistent and significant tension and restlessness. It has a global incidence of 3–25%, and the incidence in chronic diseases, such as cardiocerebrovascular disease, irritable bowel disease (IBD), cancer is 1.4–70% (Remes et al., 2016) leaving a serious impact on patients and society. Therefore, the treatment of anxiety is of utmost importance. The gut microbiota has been implicated in anxiety and studies have demonstrated that regulating the gut microbiota can effectively improve anxiety symptoms in germ-free animal models with anxiety-related behaviors (Neufeld et al., 2011). Although the probiotic interventions to regulate the gut microbiota have been found to be viable, the non-probiotic interventions [supplementary of the resistant dextrin or a low FODMAP (fermentable oligosaccharides, disaccharides, and monosaccharides and polyols)] have been reported to be more effective than the probiotic interventions (Yang et al., 2019). Nonetheless, the scope of regulating the gut microbiota to improve anxiety symptoms exists. There is a need for more relevant clinical studies to establish the relationship between the improvement of anxiety symptoms and the gut microbiota.

### Schizophrenia

Schizophrenia (SCZ) is a complex neuropsychological disorder associated with disordered thought and behavior, delusions, relapsing episodes of psychosis, and hallucinations, thus impairing daily function and social interaction. It is compounded by the interaction of genetic vulnerability with pre- and postnatal environmental factors (Socała et al., 2021). The advent of NGS technologies, especially the whole-genome analysis, has made it possible to suggest possible causes of SCZ and one such possibility is that genes related to immunity may be changed in SCZ patients (Zhu et al., 2017). The study by Song et al. (2013) found an increased immune response and inflammatory state in patients with schizophrenia. Microbiota and intestinal mucosal cells have the ability to regulate the alterations in immune-responsive chemical factors viz. anti-inflammatory interleukin-10 (IL-10) and transforming growth factor-β (TGF-β) or the pro-inflammatory IL-8 and IL-1. In comparison to the normal controls, the concentration of pro-inflammatory cytokines in SCZ patients is higher. There is also a positive correlation between the levels of inflammatory markers in serum and clinical symptoms of SCZ patients (Hope et al., 2013). Additionally, helper T cell type 1 (Th1) to Th2 ratio is higher than normal, indicating an enhanced inflammatory response in the SCZ patients (Kim et al., 2004).

The gut microbiota also regulates brain-derived neurotrophic factor (BDNF) and *N*-methyl-D-aspartate (NMDA) receptors. These molecules have a vital role in cognitive function, retention of memory, and other functions of brain (Zhu et al., 2017). BDNF and NMDA receptors play a role in neural plasticity and brain development. Alterations in the expression of BDNF lead to the cognitive dysfunction in SCZ patients, and the symptoms of schizophrenia are also provoked by the antagonists of the NMDA receptor. When the functioning of the NMDA receptor is enhanced, the symptoms of SCZ may be relieved with improved cognitive ability (Coyle, 2012). The role of gut microbiota in SCZ has been extensively reviewed elsewhere (Patrono et al., 2021) wherein the authors have discussed the possible ways to manipulate GABA production by gut microbiota, ultimately leading to SCZ.

Zhu et al. (2020) studied the association of gut microbiota and SCZ using metagenomics and found that the gut microbiota in SCZ patients harbored *Alkaliphilus oremlandii*, *Enterococcus faecium*, *Cronobacter sakazakii/turicensis*, and *Lactobacillus fermentum*. These facultative anaerobes are rare in the gut of a healthy individual. The study also indicated a close association between the gut and the oral microbiota in SCZ as the bacteria inhabiting the oral cavity, such as *Bifidobacterium dentium*, *Dialister invisus*, *Lactobacillus oris*, *Streptococcus salivarius, Veillonella atypica*, and *Veillonella dispar* were found to be more abundant in SCZ patients as compared to healthy controls (HCs).

### Autism spectrum disorder

Autism spectrum disorder is a neuropsychiatric disorder that is characterized by impaired social communications and interactions, stereotyped behaviors, and cognitive inabilities (El-Ansary et al., 2020). The term ‘spectrum’ is used to indicate the severity and type of symptoms; patients with the mild symptoms being able to function without any external support, whereas those having severe symptoms need significant support to function. The link between ASD and gut microbiota comes from the observation that approximately 70% of autistic patients exhibit comorbid disturbances in GI such as constipation, bloating, and diarrhea, providing an indication that ASD is connected with disturbances in the gut-brain axis (Stephen et al., 2017). GF mice have been found to have social behavior deficits and increased repetitive behavior (Desbonnet et al., 2014; Luczynski et al., 2016) emphasizing the requirement of an appropriate composition of the gut microbiota for the normal social development. A landmark study by Sharon et al. (2019) carried out the transplantation of the gut microbiota from autistic human donors into GF mice that resulted in the colonization of microbiota followed by the induction of autistic behavior in the mice confirming the linking etiology between autism and the gut microbiota.

Moreover, some specific microbial communities inhabiting the gut that are susceptible to broad-spectrum antibiotic vancomycin have been associated with ASD (Kim et al., 2017). In a pilot study, autistic children were treated with this antibiotic and a manifest improvement in behavioral symptoms was observed (Sandler et al., 2000). Owing to the threat of antimicrobial resistance (AMR) and its repercussions, antibiotics are not considered a feasible intervention strategy in the long-term management of any non-bacterial infection or disease including ASD (Talat et al., 2022), but this study provided a critical understanding into the potential role of gut microbiota as contributors to the behavioral disturbances in ASD. Also, the gut microbiota of autistic children showed an increased abundance of pathogenic *Clostridia* and *Desulfovibrio* genera, and a decreased abundance of the beneficial *Bifidobacterium* (Cryan et al., 2019).

As mentioned above, several studies hitherto have reported the gut microbiota alterations in autistics subjects, but there is paucity of experimental proof in favor of manipulating the gut microbiota as a treatment intervention for ASD. In an attempt to address this issue, a small open-label study validated the efficacy of fecal microbiota transplantation (FMT) of a standardized microbial cocktail to autistic children in improving the behavioral and GI symptoms (Kang et al., 2017). Leveraging the gut microbiota, a prebiotic intervention study reported that a concoction of the prebiotic B-GOS along with the casein/gluten-free diet resulted in an improved behavioral symptoms of children with ASD (Grimaldi et al., 2018). The study reported an increase in the relative abundance of the beneficial bacteria, *Bifidobacterium longum*, in the microbiota of autistic children. However, there is a pressing need for more rigorous study designs, larger sample size studies, and longitudinal research in larger cohorts.

### Bipolar disorder

Bipolar disorder is a serious neuropsychological illness defined by intense changes in mood that include emotional highs (mania or hypomania) and lows (depression) and is hypothesized to be associated with influence of the gut microbiota (Bengesser et al., 2019). Patients with BD exhibit an increased frequency of GI illnesses such as IBD, which mechanistically has been linked to gut microbiota function (Flowers et al., 2020). BD, a potent source of disability, affects 1% of the world’s population and is associated with higher rates of cardiovascular diseases, substance abuse, and significant psychosocial and functional impairment along with a high risk of mortality from suicide (Post, 2005; Alonso et al., 2011; Pompili et al., 2013; Whiteford et al., 2013).

In a study on the stool microbiome, Evans et al. (2017) found significant differences in gut microbiota between the BD patients and HCs. The BD patients showed a decreased relative abundance of *Faecalibacterium*, a prevalent gut microbe, as compared to HCs. Lower levels of *Faecalibacterium* are associated with medical conditions such as IBD (Sokol et al., 2009) and depression (Jiang et al., 2015; Aizawa et al., 2016). This microbe is reported to have anti-inflammatory properties (Sokol et al., 2008) indicating a potential therapeutic avenue for BD.

Given the fact that this microbe has useful properties and that its relative abundance is decreased in a diseased state, it may prove to be a potential psychobiotic. It is imperative to look for strategies that may be employed to mitigate the effects of BD. One such way is the implementation of supplement or nutritional strategies aimed at therapeutically increasing the beneficial microbes, such as *Faecalibacterium*, in BD patients that may improve mood symptoms, but prospective clinical trials are needed to test the viability of such strategies. Dickerson et al. (2018) reported a reduction in the rate of re-hospitalization of patients in a randomized controlled trial who were recently discharged following hospitalization for BD leveraging the probiotic therapy.

### Attention deficit/hyperactivity disorder

Attention deficit/hyperactivity disorder is one of the most common neurodevelopmental disorders that presents as difficulty in focus and organization (inattention), impulsivity, hyperactivity, and cognitive impairment (American Psychiatric Association, 2013). With a prevalence of 5.9% worldwide (Willcutt, 2012), it imposes huge social and psychological burdens on patients and families. The gut microbiota has been implicated in ADHD and as such several studies have investigated the role of the gut microbiota and the changes it undergoes in ADHD patients and HCs. Wang et al. (2022) reported an elevation in the genus *Blautia* while studying the relative abundance of gut microbiota in ADHD patients compared with HCs. In a similar study on a small clinical cohort of patients presenting with ADHD, there was an increase in the genus *Bifidobacterium* compared to HCs (Aarts et al., 2017). The modulation in the gut microbiota may open up avenues for microbiota-based interventions in ADHD. Promisingly, a probiotic supplementation with *Lactobacillus rhamnosus* during the first 6 months of life reduced the risk for ADHD diagnosis later in childhood (Pärtty et al., 2015), reflecting its potential as a possible intervention in ADHD. However, there is a pressing need for further clinical studies followed by an in-depth analysis of the resultant data. The confounding factors like the sample sizes, geographical location, diagnostic heterogeneity of ADHD, medication use, dietary patterns, sequencing, and analysis pipelines should also be taken into consideration.

## Neurodegenerative diseases and the microbiota-gut-brain axis

### Alzheimer’s disease

Alzheimer’s disease is the most common, irreversible and chronic brain disorder in which progressive degeneration of neurons leads to cognitive decline and impairments in the memory, mostly in the elderly (Wimo et al., 2017). It is the 5th leading cause of death in individuals over 65 years of age (Bulgart et al., 2020). AD patients exhibit serious behavior-, learning-, and memory impairments, severely influencing their daily activities. This neurodegenerative disorder is characterized by the death of neurons, progressive synaptic failure, deposition of amyloid-β (Aβ) around the neurons, and hyper-phosphorylated tau protein (or τ protein) aggregation in neuronal dendrites and axons (Braak and Braak, 1991; Hampel et al., 2010; Kowalski and Mulak, 2019; The Alzheimer’s Association, 2022).

Alzheimer’s disease process is believed to be initiated by the accumulation of the Aβ peptide in the brain, 15–20 years before the clinical symptoms of AD occur (Panza et al., 2019). Aβ peptide is the main component of the plaques found in AD patients (Spitzer et al., 2016). These plaques are formed as a result of invasion of brain by different pathogenic microorganisms such as HSV-1, CMV, *Borrelia burgdorferi*, *Porphyromonas gingivalis*, etc. (Fulop et al., 2018). This infection hypothesis that was once discarded by the scientific community and has gained traction of late shows that microbes might have an important role in the development of AD. The pioneering work of Itzhaki’s group found remnants of HSV-1 viral DNA in the Aβ plaques (Itzhaki, 2016). Studies have reported that other viruses, such as CMV, may also be involved in the pathogenesis of AD (Lövheim et al., 2018). Fulop et al. (2018) reviewed the nature and mode of action of plaques found in AD patients and came to a conclusion that, “in view of our infection hypothesis it can be said that the plaques in the brain of AD patients and even in the earlier stages may actually be biofilm.” This hypothesis has given a new direction to studying AD and has opened up avenues for treating AD.

As mentioned above, bacterial pathogens that infect humans have been implicated in AD, even those infecting the organs located away from the brain. In a study by Dominy et al. (2019), oral *Porphyromonas gingivalis* infection in mice resulted in colonization of the brain. This pathogen is a keystone species for the development of chronic periodontitis in the oral cavity. The inhibitors against the toxic proteases, gingipains, produced by this pathogen resulted in a reduction in the bacterial load in brain, reduced the inflammation in neurons, blocked the production of Aβ plaques, and rescued the hippocampal neurons. The study suggested that the inhibitors of gingipain proteases could be essential for treating brain colonization of *P. gingivalis* and neurodegeneration in AD.

Although there has been much disappointment in the hunt for new drugs for AD over the past decade, some studies have stimulated excitement on gut microbiota having a possible significant role in AD pathogenesis (Friedland, 2015; Itzhaki et al., 2016) and thus a possibility of targeting the gut microbiota for treating AD. Nagpal et al. (2019) reported higher abundance of Firmicutes and Proteobacteria (enterobacteriaceae) and lower abundance of Bacteroidetes in patients with mild cognitive impairment (MCI). There are reports of reduced diversity and low Firmicutes: Bacteroidetes ratio in patients with AD (Vogt et al., 2017), and lower Bacteroides in patients with dementia (Saji et al., 2019), while lower diversity, higher abundance of Proteobacteria and lower abundance of Firmicutes were reported in patients with AD compared to MCI patients and normal subjects (Liu et al., 2019). In addition to the modulation in the gut diversity, the role of microbial metabolites is also peculiar in AD. The gut microbiota metabolites exert their affects in AD in one of the two ways; either they send signals to the brain by acting on the local neuronal cells in the gut and nearby tissues, or they are absorbed from the gut and reach the brain through systemic circulation where they affect the brain functioning. These metabolites include GABA, monoamines, SCFAs, BDNF, beta-methylamino-L-alanine, dopamine, and serotonin (Bhattacharjee and Lukiw, 2013; Ho et al., 2018).

The need of the hour is to prevent or slow down AD progression and/or ameliorate its effects. Given the emerging data on disparities in the gut microbiota in AD patients as compared to healthy individuals, the scientific community has started exploring ways to ameliorate AD pathology by modulating the microbiome. There are various ways to manipulate the gut microbiota viz. using the prebiotics, probiotics, antibiotics, and synbiotics or modulations in diet, however, diet remains the primary modulator of the gut microbiota (Kincaid et al., 2021). Modulations in the diet focused on polyunsaturated fatty acids (PUFAs), whole grains, fruits and vegetables, which in turn modulate the gut microbiota, confers benefits on cognitive health in AD patients. For example, Mediterranean diet (MD) has been correlated with reduced brain atrophy in key-AD areas displaying a positive effect on pathology of AD (Mosconi et al., 2014). MD is also reported to decrease inflammation, a major symptom of AD, *via* decreasing the levels of plasma C-reactive protein and increasing carotenoids (Blum et al., 2006). Szczechowiak et al. (2019) reported that the higher consumption of docosahexaenoic acid- (a type of n-3 PUFA) and vitamin D3-rich fish products is associated with lower AD risks. Also, vitamin D-rich dairy products are involved in promoting neural growth factor protein, which protects against aging and inflammation of brain (Brown et al., 2003).

As the number of AD patients is rising rapidly worldwide, and there is no cure available till date, therefore there is a pressing need for interventions such as effective dietary modulations for AD-related inflammation and to slow down or prevent the progression of AD. The key lies in establishing the linking etiology between the gut microbiota, dementia, lifestyle, and diet. It would expose the mechanisms underlying the pathology of AD which may unravel novel intervention strategies for preventing the progression of AD or its treatment.

### Parkinson’s disease

Parkinson’s disease is a severe neurodegenerative disorder defined by the rigid movements, tremor, and abnormal gait. PD-related motor symptoms are often preceded by non-motor problems including GI function dysregulation which is manifested as nausea, bloating, gastroparesis, constipation, or weight loss (Cloud and Greene, 2011; Sung et al., 2014; Kim and Sung, 2015). The advanced stage of the disease involves characteristic brain pathology and motor impairment and at this stage a significant proportion of the dopaminergic neurons in the cells involved in motor control (substantia nigra) have degenerated axons or have been lost (Cheng et al., 2010).

Evidence from a number of research groups (Hasegawa et al., 2015; Keshavarzian et al., 2015; Sampson et al., 2016; Bedarf et al., 2017; Hill-Burns et al., 2017; Aho et al., 2019; Romano et al., 2021; Lubomski et al., 2022; Zhu et al., 2022) has emerged proposing a relationship between the gut microbiota and PD. The study by Killinger et al. (2018) proposed that the normal human vermiform appendix harbors pathogenic forms of α-synuclein that affect the risk of developing PD and that an appendectomy could be a potential prophylactic for initiation of PD. Such studies propose that the gut could be a major source of inflammation contributing to neurodegeneration in PD. In addition to an alteration in the profile of gut microbiota, there is a shift to a pro-inflammatory state that may have harmful effects in the brain and gut (Perez-Pardo et al., 2019).

α-synuclein protein aggregate, the hallmark of PD pathology, has also been identified in the sub-mucosal and mucosal nerve fibers and ganglia of PD patients (Forsyth et al., 2011; Hilton et al., 2014). Holmqvist et al. (2014) demonstrated the preclinical evidence that Parkinson’s pathology spreads from the GI tract to the brain and that α-synuclein can be transported from the gut to the brain *via* the vagus nerve which acts as a conduit for signals in the gut-brain axis.

One of the most remarkable studies that links the vagus nerve and PD was carried out by Svensson et al. (2015). They suggested that the vagal nerve may be critically involved in PD pathogenesis by demonstrating that a full truncal vagotomy is associated with a decreased risk for subsequent PD. This finding is corroborated by another study in which truncal vagotomy and α-synuclein deficiency in mice prevented gut-to-brain propagation of α-synucleinopathy and the related behavioral deficits and neurodegeneration (Kim et al., 2019). Furthermore, when the gut microbiota of PD patients was used to colonize the mice *via* FMT, they developed hallmark symptoms of PD including neuroinflammation and motor deficits (Sampson et al., 2016). This study implicated SCFAs as drivers of neuroinflammation in Parkinsonian mice. The studies on human subjects have been contrary to such findings. Data from clinical studies have showed a reduction in SCFAs in PD patients (Unger et al., 2016). Another study reported reductions in fecal SCFAs but increased plasma SCFAs in patients with PD and these changes correlated to specific gut microbiota changes and the clinical severity of PD (Chen et al., 2022). The study by Rosario et al. (2021) reported an increased capability of gut microbiota to degrade host glycans and mucin which is associated with disease severity, and established microbial contribution to hyperhomocysteinemia and folate deficiency observed in PD patients using metagenomics and metabolomics. Qian et al. (2020) used shotgun metagenomics to investigate the gut microbiota genes in PD patients and their healthy spouses, and identified twenty-five gene markers that distinguished PD patients from HCs. The authors reported that the PD index based on the gene set from the gut microbiota may be a potential diagnostic biomarker of PD.

The studies on human subjects (Hasegawa et al., 2015; Keshavarzian et al., 2015; Scheperjans et al., 2015; Unger et al., 2016; Bedarf et al., 2017; Hill-Burns et al., 2017; Heintz-Buschart et al., 2018) have been entirely cross-sectional with most of them carried out on small cohorts. There is a pressing need for larger, longitudinal, and more mechanistic studies to establish the evidence from small cohort studies. These prospective studies will also help to understand how alterations in the gut microbiota can evoke the motor and non-motor symptoms of PD and whether the profile of gut microbiota alters throughout PD progression.

### Multiple sclerosis

Multiple sclerosis is an immune-mediated and CNS-related neurological disorder characterized by aggravating motor deficits, blurry vision, and sensibility modulations appearing spontaneously with hardly any prodromal signals (Dendrou et al., 2015). Gut microbiota has been demonstrated to be essential for the development and maturation of the immune system and thus it has implications in MS pathogenesis. To put it in perspective, FMT from MS patients resulted in the mice exhibiting autoimmune encephalomyelitis (AE), one of the hallmark symptoms of MS (Berer et al., 2017; Cekanaviciute et al., 2017). This was further corroborated by studies that demonstrated GF mice showing resistance to developing AE in a MS mouse model, which was promoted by segmented filamentous gram-positive bacteria in the gut (Lee et al., 2011; Berer et al., 2017).

Multiple sclerosis generally starts as relapsing remitting form of MS (RRMS), but often shifts into secondary progressive MS (SPMS). The characteristics of the gut microbiome in different stages of MS revealed that the gut microbiome is differentially altered in these different stages. In particular, reduced SCFA biosynthesis in RRMS and elevated oxidative level in SPMS were characteristic of MS (Takewaki et al., 2021). MS-induced microbiota alterations include decreases in *Butyricimonas* and increases in *Methanobrevibacter* and *Akkermansia* (Jangi et al., 2016). In a different study, Rosario et al. (2021) found abundance levels of *Akkermansia muciniphila* and *Methanobrevibacter smithii_2* significantly increased in PD which is similar to the changes observed in MS. Thus, there is a possibility of co-occurrence of MS and PD and the same has been the subject of various studies (Nielsen et al., 2014; Thormann et al., 2017). More work is needed to unravel this relationship between MS and PD that may open-up new horizons in the treatment of such grain disorders.

*Akkermansia muciniphila* and *Acinetobacter calcoaceticus* have been found to be present in fecal samples from MS patients in a relatively higher abundance (Cekanaviciute et al., 2017). The MS patients have also been found to exhibit decrease in anti-inflammatory *Parabacteroides distasonis*.

Also, the functional potential and taxonomy of the gut microbiome has been found to differ between clinical subjects with pediatric-onset MS against controls, including depletion of the lactate fermentation pathway and higher prevalence of a methane-producing pathway from Archaea indicating that the gut microbiome of MS patients may have a disturbed functional potential (Mirza et al., 2022). Therefore, it is evident that the gut microbiota has a distinct role in MS expression, possibly controlled by interventions in the diet. Precise recommendations regarding a specific dietary plan in MS patients do not exist hitherto. However, there are reports of diet-induced microbiota changes resulting in the manifestation of experimental autoimmune encephalomyelitis (Libbey et al., 2018). Conversely, a balanced diet is associated with improvement in several clinical parameters for MS patients. There is a need for studies targeting the microbiota interventions to validate such hypotheses. To establish the role of diet in MS management, large, well-scheduled clinical trials backed by molecular, metagenomic, and metabolomics technologies are indispensable.

## Role of microbial and dietary intervention in brain disorders

### Fecal microbiota transplantation

Fecal microbiota transplantation involves the manual transfer of gut microbiota from one individual to another and is commonly performed *via* colonoscopy in humans or oral administration of freeze-dried material in rodents. Although an age-old practice, the use of FMT in human medical treatment has gained traction of late, particularly in treating neural conditions such as anxiety and depression. It has opened new horizons for more mechanistic investigations into the role of gut microbiota in various neuropsychiatric and neurodegenerative conditions viz. ASD, Schizophrenia, Alzheimer’s, Parkinson’s, Multiple Sclerosis, etc. Restoration of the balanced gut microbiota is a promising treatment for GI dysbiosis-driven brain disorders and may improve the health conditions of patients suffering from such brain disorders.

The advantages of FMT include the absence of significant side effects and safety, even in high-risk patients (Wimo et al., 2017; Haikal et al., 2019). An *in vitro* cultured gut microbial transplant (GMT) was found to considerably alleviate the anxiety-like behavior in an autistic mice (The Alzheimer’s Association, 2019). In a clinical study on ASD patients, Kang et al. (2017) validated the efficacy of FMT of a standardized microbial cocktail to autistic children in improving the behavioral and GI symptoms. A study on AD mouse model demonstrated that there exists a link between the modulation in gut microbiota composition and the cognitive dysfunction, and FMT was found to be effective in alleviating the cognitive dysfunction in AD (Reitz, 2012). However, longitudinal studies followed by larger scale clinical trials focusing on the efficacy of FMT in treating such neurological disorders will be a game changer in this regard.

### Probiotics

Probiotics are live microorganisms which when administered in appropriate amounts confer a health benefit on the host (Vallianou et al., 2020). Probiotics are gaining popularity for their use in common foods and also as pills (Draper et al., 2017). Mainly comprising of *Bifidobacterium* and *Lactobacillus* bacteria, probiotics from other genera such as *clostridium* and *Bacillus* have also been reported (Manhar et al., 2015, 2016). Studies focused on metabolites of probiotics have established them as essential mediators of host-microbiota interactions. Gut microbiota such as *Bifidobacterium*, *Ruminococcus*, *Clostridium*, *Lactobacillus*, *Bacteroides*, and *Peptostreptococcus* produce tryptophan catabolites such as tryptamine, indoleacetic acid (IAA), indole, 3-methylindole, etc. (365–370). These catabolites have been demonstrated to affect the health of the host by exerting anti-inflammatory effects and possibly by modulating the gut microbiome (64, 371). Studies on evaluating the effects of probiotics have been mostly focused on animal models where they have revealed to be beneficial for neurological disorders such as AD and ASD, and have led to improvement in the cognitive outcomes of these animal models (Cheng et al., 2019). In a clinical study on autistic children aged from 3 to 12 years, complaining of GI symptoms and having anxiety, the administration of visbiome, a probiotic product which contains eight strains of probiotics, including *Lactobacillus acidophilus* DSM24735™, *L. paracasei* DSM24733™, *Streptococcus thermophilus* DSM24731™, *L. plantarum* DSM24730™, *L. helveticus* DSM24734™, *Bifidobacterium lactis* DSM24736™, *B. breve* DSM24732™, and *B. lactis* DSM24737™ was found to be safe, and those patients who retained *Lactobacillus* reported health improvements in the GI symptoms and ASD (Arnold et al., 2019). However, there is paucity of such studies regarding the effectiveness of probiotic administration in treating neurological disorders in humans (378). More studies with large scale clinical trials are needed to establish the effectiveness of probiotics in human neuropsychiatric and neurodegenerative diseases.

### Prebiotics

International Scientific Association for Probiotics and Prebiotics (ISAPP) defines prebiotics as “a substrate that is selectively utilized by host microbes conferring a health benefit.” Prebiotics, as an alternative to probiotics, can be used to regulate the gut microbiota and, therefore, affect the health of the GI tract (Hill et al., 2014).

Prebiotics consist of soluble and fermentable fibers, human milk oligosaccharides (HMOs), and non-digestible Fructo-oligosaccharides (FOS), galacto-oligosaccharides (GOS), and trans-galacto-oligosaccharides (TOS) (Davani-Davari et al., 2019). The prebiotic therapies have been evaluated and found to have a role in enhancing beneficial host-bacteria such as *Lactobacilli* and *Bifidobacteria*, however, only a few studies have evaluated their beneficial impacts on gut microbiota in humans. Prebiotics such as FOS and GOS have been demonstrated to have anxiolytic and anti-depressant impacts, and capability of reversing the chronic stress effects (Burokas et al., 2017). Ghanizadeh and Moghimi-Sarani (2013) pursued a randomized double blind placebo-controlled clinical trial and found that the administration of N-acetylcysteine resulted in a decline in repetitive behavior and irritability in autistic patients. In another study, supplementation of B-GOS prebiotic along with a restrictive diet led to the improvement in the behavior of autistic children and was hypothesized to be associated with *Bifidobacterium* and *Lactobacillus* abundance (Grimaldi et al., 2017). The above findings demonstrate that prebiotics can be potential treatment option for various neurological disorders. However, more studies are needed to better understand the underlying mechanisms of prebiotic action and establish the role of prebiotics in brain disorders.

### Dietary interventions

One of the most active areas of research in deciphering the possible treatment of brain disorders is the study of the role of diet on brain functioning. Several dietary components viz. Omega-3 fatty acids (e.g., docosahexanoic acid), curcumin, flavonoids, B-vitamins, saturated fats, Vitamin D, Calcium, Selenium, Zinc, Choline, etc., have been found to effect cognitive abilities (Gómez-Pinilla, 2008).

Omega-3 fatty acids–rich diet has been demonstrated to be involved in strengthening cognitive processing in humans (McCann and Ames, 2005) and up-regulating genes necessary for maintaining plasticity and synaptic function in rats (Wu et al., 2007). A deficiency of omega-3 fatty acids led to impaired memory and learning in rats (Bourre et al., 1989; Moriguchi et al., 2000). Diets also have adverse effects on cognitive processing when they contain undesired components. For example, diets rich in saturated fats hamper molecular substrates necessary for supporting cognitive processing and thus increase the risk of neurological dysfunction in humans (Greenwood and Winocur, 2005) and rats (Molteni et al., 2002). Animal studies evaluating the effects of diet rich in saturated fat have shown reduced hippocampal levels of BDNF-related synaptic plasticity and cognitive performance (Molteni et al., 2002).

Like Omega-3 fatty acids, research has demonstrated that other nutrients viz. curcumin, flavonoids, selenium, zinc, vitamins, etc., are vital for cellular processes and molecular systems that are essential for maintaining cognitive function (Gómez-Pinilla, 2008). This raises an exciting possibility of using dietary interventions as a potential strategy for enhancing cognitive processing. Moreover, there is also a possibility of modulating the gut microbiota by dietary interventions, which in turn may lead to enhancement in the brain disorders. A study by So et al. (2018) observed the effect of fiber on the gut microbiota in a review and meta-analysis from 64 studies. They observed an increase in the fecal abundance of *Lactobacillus* and *Bifidobacterium* species on dietary fiber interventions, particularly fructans and GOS.

Although these studies demonstrate effects of diet on the brain functions, more research is needed to determine the underlying mechanisms of action of diets and the conditions for therapeutic applications in human diseases.

## Conclusion

Hippocrates is believed to have said: “let thy food be thy medicine and thy medicine be thy food” (Cryan et al., 2019). After decades of research on the microbiota-gut-brain axis, a modified version now seems appropriate “let food for thy gut microbiota be medicine for thy brain.”

The connection between gut-brain axis and gut microbiota has been rendered to substantial research over the last 10 years or so. The gut microbiota is believed to be critical for the normal development and maintenance of functions of the brain. Further, there is accruing evidence from studies on animal models as well as clinical subjects involving the modulations in gut microbiota in a plethora of neurological, psychiatric, and neurodegenerative disorders (Table 1). However, the successful treatment of such disorders is yet to be achieved and is expected to manifest in improvement in clinical cohorts in placebo-controlled trials.

**TABLE 1:** Changes in gut microbiota in various neuropsychiatric and neurodegenerative diseases.

Disorder	Symptoms	Changes in gut microbiota	References
Depression and anxiety	Persistent and significant tension and restlessness	↓ Bifidobacterium and lactobacillus ↑ Bacteroidetes, Proteobacteria, and Actinobacteria ↓ Firmicutes ↓ Coprococcus and Dialister	Jiang et al., 2015; Aizawa et al., 2016; Valles-Colomer et al., 2019
Schizophrenia	Psychosis, delusion, cognitive dysfunction, Apathy, social withdrawal	↑ Bacteroidetes and Actinobacteria ↑ *Streptococcus gordonii*, *S. thermophilus*, and *Streptococcus* sp. (oral taxon 071) ↑ Candida ↑ Eubacterium ↓ Neisseria, Haemophilus, and Capnocytophaga	Castro-Nallar et al., 2015
Autism spectrum disorder	Impaired social communications and interactions, stereotyped behaviors, and cognitive inabilities	↑ Clostridia and Desulfovibrio ↓ Bifidobacterium ↑ Bifidobacterium longum	Grimaldi et al., 2018; Cryan et al., 2019
Bipolar disorder	Emotional highs (mania or hypomania) and lows (depression).	↓ Faecalibacterium	Evans et al., 2017
Attention deficit/hyperactivity disorder	Inattention, impulsivity, hyperactivity, and cognitive impairment	↑ Blautia ↑ Bifidobacterium	Aarts et al., 2017; Wang et al., 2022
Alzheimer’s disease	Cognitive decline, impairments in the memory, behavior and learning	↑ Firmicutes and Proteobacteria ↓ Bacteroidetes ↓ Firmicutes ↑ Bacteroidetes ↓ Bifidobacterium	Vogt et al., 2017; Nagpal et al., 2019
Parkinson’s disease	Rigid movements, tremor, and abnormal gait	↓ Prevotellaceae ↑ Enterobacteriaceae ↑ Lactobacillus ↓ *Clostridium coccoides* ↓ *Bacteroides fragilis*	Hasegawa et al., 2015; Scheperjans et al., 2015
Multiple sclerosis	Motor deficits, blurry vision, and sensibility modulations	↓ Butyricimonas ↑ Methanobrevibacter and Akkermansia ↑ *Akkermansia muciniphila* and *Acinetobacter calcoaceticus* ↓ *Parabacteroides distasonis*	Jangi et al., 2016; Cekanaviciute et al., 2017

There is a pressing need for large and longitudinal clinical trials to establish that the alterations in the gut microbiota profile are fundamental to the pathophysiology of such disorders. There seems to be a shift from microbiota-gut-brain axis to diet-microbiota-gut-brain axis as there is growing evidence that diet plays a pivotal role in shaping the diversity of the gut. So more studies in the future should be carried out in order to establish the therapeutic potential of diet-based modulations of gut microbiota and it might prove to be the conduit for future personalized-medicine approaches.

## Author contributions

YB wrote whole review manuscript. AK conceived idea and guided and checked draft manuscript. Both authors contributed to the article and approved the submitted version.
